# Supplementary material for: Evaluation of protection benefit of sand barrier fence with different heights on desert highway
Source: PLoS One. 2025 Aug 8;20(8):e0324869. doi: 10.1371/journal.pone.0324869 (PMC12334077; doi:10.1371/journal.pone.0324869)
Supplement: Attachment — Submitted filename: Manuscript revision reply letter.docx [file pone.0324869.s002.docx]

**Manuscript revision reply letter**

Dear editor:

First of all, thank you very much for taking the time to read and evaluate my manuscript carefully in your busy work. Your valuable comments and suggestions are of great significance to me. Secondly, in your letter, I noticed that you have put forward some opinions and suggestions on the format of my manuscript. I have further modified the format of the paper to make it conform to the requirements of the format of your paper. At the same time, if there are other problems in the paper, I hope you can tell me that I will modify and reply in time, and hope that the manuscript can be published in your journal as soon as possible. Finally, I wish you and the review experts a happy life and a smooth work ! Thank you again !

**Reviewer1**

1.There are some abbreviations that must be translate (UAV) and (HDPE).

Reply : Thank you for your valuable comments.The translation abbreviations have been corrected.

2.The simulation grid description is very confused. They should make clear for reader. The images are good, but the description is not.2）

Reply : Thank you for your valuable comments, I am very sorry that due to space constraints did not elaborate on the meshing process. The grid division of this study is carried out directly in ICEM. In order to ensure the quality of the grid, the block processing method is adopted. Considering that the hexahedral mesh has high quality and small calculation error, the hexahedral mesh is used in the modeling process. The sand particles in the wind-blown sand flow are more in the near-surface height range and the distribution is more complex than that in the upper layer. At the same time, the boundary layer has a great influence on the wind-sand incoming flow. Therefore, the near-surface position of the model is encrypted during the meshing process, and the position near the top surface of the model is sparsely divided to save computing resources and speed up the operation. The meshing has now been re-described, as shown in line P3 13-21 .

3. Did the authors test the mesh quality, or was the mesh chosen primarily based on simulation time considerations? Additionally, there appears to be some confusion between the terms "grid" and "cell." Clarification and consistency in the usage of these terms are needed.

Reply : Thank you for your valuable comments. In numerical simulation, the quality of the grid and the choice of the grid are both crucial factors. The quality of the grid directly affects the accuracy of the calculation results. This paper considers two aspects. On the one hand, the quality of the grid usually involves the division of the grid, the density of the grid, the shape of the grid, etc. In this paper, the grid is structured and divided into hexahedrons.

On the other hand, the choice of grid also takes into account the simulation time. The density and number of meshes will directly affect the amount of calculation, which in turn affects the time required for simulation.

Therefore, in order to ensure the calculation accuracy and save the calculation cost, the grid is encrypted at the position where the flow field changes strongly, such as near the wall surface and the height range of the protection system. However, the flow field changes more stably at other positions in the fluid domain, and the grid size is set larger. Finally, the grids of each part are spliced.

There appears to be some confusion between the terms ' grid ' and ' cell ', which has been corrected.

4. There is insufficient description of the conditions under which the fluid and particles interact. While this may be implicit in the CFD method, it is important to clearly define these conditions. How do the authors define sand accumulation, particularly if the model used is based on a two-phase approach?

Reply : Thank you for your valuable comments.The Euler model takes a different perspective. It regards the continuous medium of each phase as an intertwined whole, and needs to solve the momentum equation and continuity equation of each phase independently. By calculating the interaction force between phases, the Euler model can accurately describe the distribution of each phase at different positions and moments. After comprehensive comparison and consideration, there are both mutual penetration and relative independence between gas and particles in wind-blown sand movement. Each has its own unique physical properties and volume ratio, but it is intertwined at the same time and space, forming a complex coupling movement. In order to describe and simulate this motion process more accurately, this paper decided to use the Euler two-phase flow model for simulation research.

The movement of the fluid should follow the law of conservation of physics. The gas phase and the sand phase should conform to the basic continuity equation and satisfy the law of conservation of momentum. Combined with the research object of this paper, the mathematical expression of the two-phase control equation is as follows :

The continuity equation of the gas phase :

| $\frac{\partial}{\partial t}\left(\alpha_{a}\rho_{a}\right)+\nabla\cdot\left(\alpha_{a}\rho_{a}\vec{v}_{a}\right)=0$ | (1) |
| --- | --- |

The continuity equation of sand phase :

| $\frac{\partial}{\partial t}\left(\alpha_{s}\rho_{s}\right)+\nabla\cdot\left(\alpha_{s}\rho_{s}\vec{v}_{s}\right)=0$ | (2) |
| --- | --- |

The volume fraction of two phases :

| $\alpha_{a}+\alpha_{s}=1$ | (3) |
| --- | --- |

In the formula： *ρ—*density；

*t—*time；

*v*_a_、*v*_s_*—*velocity vectors of gas phase and sand phase；

*α*_a_、*α*_s_*—*volume fraction of gas phase and sand phase；

The momentum equation of the gas phase :

| $\frac{\partial}{\partial t}\left(\alpha_{a}\rho_{a}\vec{v}_{a}\right)+\nabla\cdot\left(\alpha_{a}\rho_{a}\vec{v}_{a}\vec{v}_{a}\right)=-\alpha_{a}\nabla p+\nabla\bar{\bar{\tau}}_{a}+\alpha_{a}\rho_{a}\vec{g}+\vec{R}_{pa}+\vec{F}_{lift,a}$ | (4) |
| --- | --- |

The momentum equation of sand phase :

| $\frac{\partial}{\partial t}\left(\alpha_{s}\rho_{s}\vec{v}_{s}\right)+\nabla\cdot\left(\alpha_{s}\rho_{s}\vec{v}_{s}\vec{v}_{s}\right)=-\alpha_{s}\nabla p+\nabla\bar{\bar{\tau}}_{s}+\alpha_{s}\rho_{s}\vec{g}+\vec{R}_{ps}+\vec{F}_{lift,s}$ | (5) |
| --- | --- |

In the formula :：$\vec{g}$*—*acceleration of gravity；

$\bar{\bar{\tau}}_{a}$、$\bar{\bar{\tau}}_{s}$*—*The stress-strain tensor of two phases；

$\vec{F}_{lift,a}$*—*lift；

$\vec{R}_{pq}$*—*The interaction force between two phases。

| $\bar{\bar{\tau}}_{a}=\alpha_{a}\mu_{a}(\nabla\vec{v}_{a}+\nabla\vec{v}_{a}^{T})+\alpha_{a}(\lambda_{a}-\frac{2}{3}\mu_{a})\nabla\cdot\vec{v}_{a}\bar{\bar{I}}$ | (6) |
| --- | --- |
| $\bar{\bar{\tau}}_{s}=\alpha_{s}\mu_{s}(\nabla\vec{v}_{s}+\nabla\vec{v}_{s}^{T})+\alpha_{s}(\lambda_{s}-\frac{2}{3}\mu_{s})\nabla\cdot\vec{v}_{s}\bar{\bar{I}}$ | (7) |
| $\vec{F}_{lift}=-0.5\alpha_{s}\rho_{q}(\vec{v}_{q}-\vec{v}_{p})\times(\nabla\times\vec{v}_{q})$ | (8) |

In the formula ：*µ_a_*、*µ_s_* — shear viscosity coefficient；

λ*_a_*、λ*_s_* *—* bulk viscosity。

5. The Authors comment that “Desert wind-sand flow is generally in a saturated state”, but they do not explain what it means or use references about the subject.

Reply : Thank you for your valuable comments.Since the desert wind-sand flow is generally in a saturated state, the sand volume fraction is large, and the two-fluid model is suitable for simulation. For the Gobi wind-sand flow, it is generally in an unsaturated state, and the sand volume fraction is small, and the Euler-Lagrange method is suitable. At present, most researchers generally use the Euler two-fluid model for desert wind-sand flow .(4-7 lines on P5)

6. Is there a specific reason for using a symmetric boundary condition at the top? To be honest, I did not fully understand the sentence: “the rest surface adopts the symmetrical boundary conditions” (SYMMETRY). Could the authors clarify this point?

Reply: Thank you for your valuable comments.The upper wall of the model adopts symmetrical boundary conditions because the normal velocity on the symmetrical surface is 0, so there is no diffusion flux on the symmetrical surface, that is, in all flow variables, the normal gradient on the symmetrical surface is also 0. At the same time, the symmetrical boundary can reduce the calculation by several times under the condition of maintaining accuracy, thus greatly reducing the calculation amount. In this paper, the surface of the symmetrical boundary includes the top surface, the front and the back of the model, as shown in the following figure, and the corresponding changes have been made in the text.

7. I am confused about the coordinate’s description in eq. (5) (input velocity profile).7）

Reply : Thank you for your valuable comments, I am very sorry, the original velocity formula, the error of the three-dimensional velocity field in the z ( height direction ) expressed as y, the formula has been corrected, as shown below, the text see P5 page Formula ( 5 ).

$$\nu\left(z\right)=\frac{\nu}{k}\ln\frac{z}{z_{0}}$$

In the formula :

v(z)—Velocity at height y;

v—Friction velocity;

k—Carmen constant, generally 0.4 ;

z—Height from the surface ;

z_0_—The surface roughness, wind speed is 0 of a certain geometric height, generally take the bed surface sand average particle size of 1 / 30.

8.Could the authors provide a 3D view of the velocity field? I am particularly interested in observing the velocity field near the fence borders.

Reply：Thank you for your valuable comments. Fig.1 is the three-dimensional velocity cloud around the fence under different sand barrier heights.

|  | |
| --- | --- |
|  |  |
| (a)H_s_=2.0m | (b)H_s_=2.5m |
|  |  |
| (c)H_s_=3.0m | (d)H_s_=3.5m |
| Fig.3D velocity cloud diagram around fence under different height of sand barrier | |

9.In the section on horizontal wind speed, it is unclear whether the velocity is composed of two components along the z and x directions or if it is only a function of x. Additionally, the results presented in this section show comparative values, but it is not clear whether these values are related to the velocity field without fences or if they are being compared to the input profile.

Reply : Thank you for your valuable comments.In this paper, the velocity at the entrance of the model adopts the logarithmic flow wind speed, and the typical wind speed contour line is shown as follows. The formula shows that at the entrance, the wind speed changes with the entrance height. The wind speed in the x direction is the horizontal wind speed mentioned in the article, and its value is closely related to the height value in the z direction. The wind speed is calculated by the following formula, and the wind speed at the height of 10 m is 15 m / s.

| $\nu\left(z\right)=\frac{\nu}{k}\ln\frac{z}{z_{0}}$ | (2.5) |
| --- | --- |

In the formula :

v(z)—Velocity at height y;

v—Friction velocity;

k—Carmen constant, generally 0.4 ;

z—Height from the surface ;

z_0_—The surface roughness, wind speed is 0 of a certain geometric height, generally take the bed surface sand average particle size of 1 / 30.

10.The velocity field profiles (contour lines) shown in Figure 7 appear almost identical, with only slight differences. How, then, could the calculated efficiency values differ so significantly, given that they are derived from the velocity profile? Is there an explanation for this apparent discrepancy?

Reply : Thank you for your valuable comments.Fig.7 is the extracted cross-section velocity flow field cloud map. The adjacent solid lines in the figure are represented as isolines. It can be seen from the velocity band that the velocity interval is 1m / s, which only characterizes the general partition law of the velocity flow field, but cannot intuitively distinguish the accurate value of the velocity. The windproof efficiency calculated later in this paper is the accurate velocity value extracted by inputting specific coordinate values in the cross-sectional flow field diagram.

|  | |
| --- | --- |
|  |  |
| (a)H_S_=2m | (b)H_S_=2.5m |
|  |  |
| (c)H_S_=3m | (d)H_S_=3.5m |
| Fig.7 Flow field structure of different sand barrier heights | |

11. Table 2 raises the question of whether there are any correlations between the efficiency of the first/second barrier and the second/third barrier. For which fence height is the decrease in efficiency more pronounced? Additionally, does this depend on the distance from the bottom surface?

Reply : Thank you for your valuable advice, the airflow velocity dropped sharply after encountering the first sand-blocking fence. Compared with the initial wind speed, the decline was larger, the wind speed decreased significantly, and even decreased to negative value after the sand-blocking fence ; after the second and third sand-blocking fences, although the airflow velocity is still low, the speed recovery after the first sand-blocking fence shows an overall upward trend, even after the third sand-blocking fence, it is greater than the wind speed, and the wind-sand flow develops well, so the sand-blocking fence fails to give full play to the wind-proof effect.

It can be seen from the table that when the height of the sand-blocking fence is 2m, the wind-proof efficiency between the second and third sand-blocking fences is the largest than that between the first and second sand-blocking fences, which is reduced by 14.09 %. When the height of the sand-blocking fence is 2.5m, the wind-proof efficiency between the second and third sand-blocking fences increases, and the wind-proof efficiency between the first and second sand-blocking fences does not decrease significantly.

When the height of the sand-blocking fence is 3m, the increase in wind-proof efficiency between the second and third sand-blocking fences is not much different from that of the sand-blocking fence 2.5m, but the wind-proof efficiency between the first and second sand-blocking fences decreases. When the height of the sand-blocking fence is 3.5m, the wind-proof efficiency between the first and second sand-blocking fences decreases the most, and the wind-proof effect is not fully exerted. Therefore, in summary, compared with other heights, when the height of the sand-blocking fence is increased to 2.5m, it has a better wind-proof and sand-blocking effect.(9-19 lines on P11)

Table2 The average windproof efficiency of each position of sand barrier before and after improvement

| Height of sand barrier (m) | The average windbreak efficiency of the first to the second sand barrier (%) | | The average windbreak efficiency of the second to the third sand barrier (%) | |
| --- | --- | --- | --- | --- |
|  | 0.1m | 0.3m | 0.1m | 0.3m |
| 2 | 89.83 | 92.35 | 79.74 | 78.26 |
| 2.5 | 86.94 | 89.10 | 90.22 | 90.16 |
| 3 | 81.55 | 84.08 | 93.16 | 93.13 |
| 3.5 | 71.82 | 75.31 | 95.26 | 96.44 |

12. The authors should provide a more thorough analysis of the data presented in the tables. For instance, the data in Table 3 indicates that accumulation reaches saturation as the fence height increases. However, no discussion of these results is included in the paper.

Reply : Thank you for your valuable comments, I am very sorry for not having a comprehensive discussion of Table 3, and now the discussion of the results has been added, as shown below, corresponding to P11 28-31 lines and P12 1-5 lines.

The thickness of sand accumulation in the range of 0.55 ~ 0.6 is extracted from the sand volume fraction between three different heights of sand-blocking fences. The results are shown in Table 3. It can be seen from the table that the thickness of sand accumulation increases with the height of sand-blocking fence between the first and second sand-blocking fences and between the second and third sand-blocking fences. When the height of sand-blocking fence is 2.5m, the thickness of sand accumulation is 50.51 % and 58.33 % higher than that of 2m high sand-blocking fence. It has a better blocking effect on sand particles in the airflow, and the effect of sand-blocking is the most significant. When the height increases to 3.5m, the thickness of sand accumulation does not increase significantly.

It can be seen that with the increase of the height of the sand-blocking fence, the thickness of the sand between the first and second sand-blocking fences and between the second and third sand-blocking fences increased compared with the height of the sand-blocking fence at the site of 2m. When the height of the sand-blocking fence increases to more than 2.5m, the thickness of the sand reaches a saturated state and no longer increases. It can be seen that the height of the sand-blocking fence should not be too low. Too low will lead to the inability to effectively block the deposition of sand particles and then move forward, which will cause sand burial and other phenomena. The high windproof efficiency of the sand barrier has not been greatly improved, and it will bring the risk of being blown down by the strong wind while increasing the cost. In summary, when the height of the sand barrier is increased to 2.5 m, it is the most reasonable height and has a better wind and sand resistance effect.

Table3 The thickness of sand accumulation in the sand barrier fence is 0.55 ~ 0.6 thickness of sand accumulation in the color belt

| Height of sand barrier (m) | The Sand thickness of first and second sand-blocking fences  (m) | The Sand thickness of second and third sand-blocking fences  (m) |
| --- | --- | --- |
| 2 | 0.196 | 0.096 |
| 2.5 | 0.295 | 0.152 |
| 3 | 0.295 | 0.155 |
| 3.5 | 0.294 | 0.154 |

13. Several sentences need to be revised for clarity, as they may confuse readers.）

Reply:Thank you for your valuable comments.I have revised these sentences.

14. The authors should review all figure captions to enhance their descriptions, as captions are essential for understanding the figures.

Reply : Thank you for your valuable comments, I 've checked all the titles and made changes to the missing chart titles, as shown here.

15.We know that fence efficiency is measure in terms of transport. I think the Authors should include a conection with sand transport in terms of the shear stress treshold for the sand movment. The shear treshold depen on the grain size. There are some discussions how to introduce this connection on the following papers:

Reply : Thank you for your valuable advice. This paper mainly studies the smooth speed, horizontal wind speed and sand accumulation distribution law under different sand barrier heights, calculates its wind protection efficiency according to its horizontal wind speed, and extracts sand accumulation thickness to evaluate the protection benefit of sand barrier. It does not consider the study from the perspective of the mechanical properties of sand movement, but in the future this is also the content of our upcoming research.

**Reviewer 2:**

1.The introduction and conclusions read like a report for the Chinese government. The text mentions a lot of numbers for sand density, porosity, wind speed, etc., specifically about the model for the study area, without a clear message about what is being learned from the simulation results. Moreover, are there any insights from the simulation that could be maybe useful for combating desertification or soil erosion in other areas of the world? Overall, this paper reads like a governmental report, rather than a scientific paper.

Reply:Thank you for your valuable comments. I am very sorry that the introduction and conclusion of this paper read like a report to the Chinese government. This paper relies on the on-site protection system of the Wuma Expressway to model and construct four different height sand barrier models. The velocity flow field, horizontal wind speed, and sand distribution characteristics were calculated and analyzed. Finally, the reasonable height parameters of the sand barrier were selected, and the windbreak efficiency under the reasonable height parameters was verified, thus verifying the rationality and feasibility of the parameters.

2.One more piece of evidence that this has been a governmental report, which has been translated to English and then submitted to Plos ONE: The bibliography contains ONLY publications from Chinese authors. However, there is so much groundbreaking literature on CFD modeling of wind-blown sand transport, completely ignored by the authors. For example, Smyth (2016) wrote an outstanding review on CFD applied to this area, see here:

Reply:I am very sorry, this article may have some problems in the writing method, so that the article looks like a report article, now some places have been modified.

3.The paper overall is not well written. A thorough revision of the language is definitely required. Moreover, the model description has various flaws. I skipped some parts of it since I will only give any further consideration to this paper if the authors resubmit a version that successfully addresses the major comments raised here, which are enough to preclude publication in PLOS ONE. However, for example, equation (5) is described incorrectly. The height above the ground is "z" and not "y". Moreover, "y_0" is not the wind speed at surface, it is rather the roughness, which is the height at which the wind velocity is zero. This kind of error shows one thing: The authors did not perform a careful job. They just submitted this manuscript to see what happens. And it astounds me that the journal chosen by the authors is PLOS ONE. This is a strong interdisciplinary journal, to which we should submit papers of high quality only.

Reply:I am very sorry, there are some mistakes in the formula description, which have been corrected now.
