# Supplementary material for: Evaluation of protection benefit of sand barrier fence with different heights on desert highway
Source: PLoS One. 2025 Aug 8;20(8):e0324869. doi: 10.1371/journal.pone.0324869 (PMC12334077; doi:10.1371/journal.pone.0324869)
Supplement: Attachment — Submitted filename: reply letter.docx [file pone.0324869.s003.docx]

**Manuscript revision reply letter**

Dear editor:

First of all, thank you very much for taking the time to read and evaluate my manuscript carefully in your busy work. Your valuable comments and suggestions are of great significance to me. Secondly, in your letter, I noticed that you have put forward some opinions and suggestions on the format of my manuscript. I have further modified the format of the paper to make it conform to the requirements of the format of your paper. At the same time, if there are other problems in the paper, I hope you can tell me that I will modify and reply in time, and hope that the manuscript can be published in your journal as soon as possible. Finally, I wish you and the review experts a happy life and a smooth work ! Thank you again !

1. If the authors have adequately addressed your comments raised in a previous round of review and you feel that this manuscript is now acceptable for publication, you may indicate that here to bypass the “Comments to the Author” section, enter your conflict of interest statement in the “Confidential to Editor” section, and submit your "Accept" recommendation.

Reply : We express our heartfelt thanks to the reviewers for their detailed review work. In this revision, we have added literature from non-Chinese authors to enhance the comprehensiveness of citations.

2. Is the manuscript technically sound, and do the data support the conclusions?

The manuscript must describe a technically sound piece of scientific research with data that supports the conclusions. Experiments must have been conducted rigorously, with appropriate controls, replication, and sample sizes. The conclusions must be drawn appropriately based on the data presented.

Reviewer #2: Partly

Reply : This paper is based on Fluent software, and the data are obtained by the calculation and post-processing function of Fluent software. The calculation process and control equations strictly follow the Euler two-fluid method, and the calculation results have reached the convergence state, thus ensuring the correctness of the calculation process. In addition, the later data processing is strictly extracted and analyzed according to the calculation file, which ensures the accuracy of data processing.

3. Has the statistical analysis been performed appropriately and rigorously?

Reviewer #2: N/A

Reply : The data statistics in this paper mainly rely on Tecplot post-processing software. The cloud map and wind speed data displayed in the article are imported into the calculation data through the software and processed. The analysis process uses Origin software for scientific drawing and data analysis, and the whole process strictly follows the originality of the calculated data.

4. Have the authors made all data underlying the findings in their manuscript fully available?

The [PLOS Data policy](http://track.editorialmanager.com/CL0/http://www.plosone.org/static/policies.action%23sharing/1/010f0194396a0b30-fc444e62-286f-46bb-8ab5-fc4d36b86d90-000000/vPlaMx3giBVegvzNsv01muxPTafR262EEdGfQ3SH5II=191) requires authors to make all data underlying the findings described in their manuscript fully available without restriction, with rare exception (please refer to the Data Availability Statement in the manuscript PDF file). The data should be provided as part of the manuscript or its supporting information, or deposited to a public repository. For example, in addition to summary statistics, the data points behind means, medians and variance measures should be available. If there are restrictions on publicly sharing data—e.g. participant privacy or use of data from a third party—those must be specified.

Reviewer #2: Yes

Reply : The availability of the data covered in this article has been uploaded to the public repository in accordance with the requirements of your publication. The corresponding URL link is : https://doi.org/10.5061/dryad.76hdr7t5s

5. Is the manuscript presented in an intelligible fashion and written in standard English?
PLOS ONE does not copyedit accepted manuscripts, so the language in submitted articles must be clear, correct, and unambiguous. Any typographical or grammatical errors should be corrected at revision, so please note any specific errors here.

Reviewer #2: No

Reply : This article has undergone rigorous ' spelling proofreading ' and ' grammar proofreading ', and has been carefully polished for the text, aiming to ensure the optimization of the article structure, the correction of grammar and spelling errors, the adjustment of language expression, and the follow-up of academic norms to meet the publishing standards of your journal.

6. Review Comments to the Author

Please use the space provided to explain your answers to the questions above. You may also include additional comments for the author, including concerns about dual publication, research ethics, or publication ethics. (Please upload your review as an attachment if it exceeds 20,000 characters)

Reviewer #2: The authors ignored my comment #2, so that the cited literature remains essentially an acknolwedgment to Chinese authors working in the field. Moreover, the reply letter does not indicate any action to address my comment #1.

I thus stopped at comment #2 and concluded that the authors decided to neglect my comments.
I am recommending alternative reviewers to the journal.

Reply : We apologize for the omission of your reply in our previous reply. A supplementary comment reply is attached.

The references you mentioned earlier have been added to the introduction of this article. Please refer to the introduction for details.See lines 32-33 on the first page and lines 16-22 on the second page.

7. PLOS authors have the option to publish the peer review history of their article ([what does this mean?](https://track.editorialmanager.com/CL0/https://journals.plos.org/plosone/s/editorial-and-peer-review-process%23loc-peer-review-history/1/010f0194396a0b30-fc444e62-286f-46bb-8ab5-fc4d36b86d90-000000/ZA6uPcAOpGm9OcneeljwkkcXWqDBD9Wb8M7G1ccCNSo=191)). If published, this will include your full peer review and any attached files.

If you choose “no”, your identity will remain anonymous but your review may still be made public.Do you want your identity to be public for this peer review? For information about this choice, including consent withdrawal, please see our [Privacy Policy](https://track.editorialmanager.com/CL0/https://www.plos.org/privacy-policy/1/010f0194396a0b30-fc444e62-286f-46bb-8ab5-fc4d36b86d90-000000/B4UkIXvKJZVm-_9zahdShY4UJhNbaNkMqrARkEyJyPQ=191).

Reviewer #2: No

Reply : I decided to remain anonymous.
