# Supplementary material for: Evaluation of protection benefit of sand barrier fence with different heights on desert highway
Source: PLoS One. 2025 Aug 8;20(8):e0324869. doi: 10.1371/journal.pone.0324869 (PMC12334077; doi:10.1371/journal.pone.0324869)
Supplement: Attachment — Submitted filename: Response to Reviewers.docx [file pone.0324869.s004.docx]

**Manuscript revision reply letter**

Dear editor:

First of all, thank you very much for taking the time to read and evaluate my manuscript carefully in your busy work. Your valuable comments and suggestions are of great significance to me. Secondly, in your letter, I noticed that you have put forward some opinions and suggestions on the format of my manuscript. I have further modified the format of the paper to make it conform to the requirements of the format of your paper. At the same time, if there are other problems in the paper, I hope you can tell me that I will modify and reply in time, and hope that the manuscript can be published in your journal as soon as possible. Finally, I wish you and the review experts a happy life and a smooth work ! Thank you again !

1.Include relevant papers in the revised manuscript.

Reply : Two papers have been added to the article, as shown in line p1 33-39.

2.Grid independence test results should be added in the paper.

Reply:Because the sand-blocking measures such as sand-blocking fences in this paper are network structures, it is necessary to use the porous medium method to characterize the porosity size. The grid division is divided by array, and other division methods cannot accurately simulate porosity. Therefore, the grid independence verification in this paper is only carried out for the subgrade, and three grid division methods are adopted, which are : extracting the horizontal wind speed at a height of 0.1 m on the top of the embankment. From the Figure.4, the grid division method does not affect the wind speed. The horizontal wind speed at different positions under the three division methods almost overlaps, and the wind speed error does not exceed 1 %. Therefore, the grid division method does not affect the wind speed. It is proved that the model grid in this paper is irrelevant.

3.The boundary conditions are not clear.The surfaces which are symmetric should be clearly shown on Figure and mentioned in the relevant section of the paper.

Reply : The coordinates have been re-added and the boundary condition description is supplemented in the figure, as shown in Figure 2.

4.The properties of HDPE are not given in the paper.

Reply : The main component of HDPE raw material is high-density polyethylene, which is processed by high-strength composite material combined with special weaving process. It has strong anti-ultraviolet radiation, frost resistance, decay resistance, acid, alkali, salt corrosion resistance, long service life and other characteristics. The unique scientific design structure of the sand-proof net can not only make the net body shake with the wind, but also make it have self-purification function and avoid dust accumulation, As shown in line p3 12-17.

5.The contours in Fig. 5 are similar and the effect of fence height is not noticed. Similarly, the contours of sand thickness distribution in Fig. 8 for four cases do not differ. Better Figures are required to explain the effect of fence height.

Reply: The influence of fence height on the structure of the flow field in Figure 5 has been re-added to the magnified picture of the local low-speed vortex zone, as shown in Figure 6, and the analysis content has been added again, such as p8 16-32 lines and p9 1-2 lines.

In Fig.8, the influence of different sand barrier heights on the distribution of sand accumulation is extracted in detail in Table 3.It can be seen from the table that the thickness of sand accumulation increases with the height of sand-blocking fence between the first and second sand-blocking fences and between the second and third sand-blocking fences. When the height of sand-blocking fence is 2.5m, the thickness of sand accumulation is 50.51 % and 58.33 % higher than that of 2m high sand-blocking fence. It has a better blocking effect on sand particles in the airflow, and the effect of sand-blocking is the most significant. When the height increases to 3.5m, the thickness of sand accumulation does not increase significantly.It can be seen that with the increase of the height of the sand-blocking fence, the thickness of the sand between the first and second sand-blocking fences and between the second and third sand-blocking fences increased compared with the height of the sand-blocking fence at the site of 2m. When the height of the sand-blocking fence increases to more than 2.5m, the thickness of the sand reaches a saturated state and no longer increases. It can be seen that the height of the sand-blocking fence should not be too low. Too low will lead to the inability to effectively block the deposition of sand particles and then move forward, which will cause sand burial and other phenomena. The high windproof efficiency of the sand barrier has not been greatly improved, and it will bring the risk of being blown down by the strong wind while increasing the cost. In summary, when the height of the sand barrier is increased to 2.5 m, it is the most reasonable height and has a better wind and sand resistance effect.
