# Supplementary material for: Evaluation of protection benefit of sand barrier fence with different heights on desert highway
Source: PLoS One. 2025 Aug 8;20(8):e0324869. doi: 10.1371/journal.pone.0324869 (PMC12334077; doi:10.1371/journal.pone.0324869)
Supplement: Attachment — Submitted filename: Response_to_Reviewers_auresp_4.docx [file pone.0324869.s005.docx]

**Manuscript revision reply letter**

Dear editor:

First of all, thank you very much for taking the time to read and evaluate my manuscript carefully in your busy work. Your valuable comments and suggestions are of great significance to me. Secondly, in your letter, I noticed that you have put forward some opinions and suggestions on the format of my manuscript. I have further modified the format of the paper to make it conform to the requirements of the format of your paper. At the same time, if there are other problems in the paper, I hope you can tell me that I will modify and reply in time, and hope that the manuscript can be published in your journal as soon as possible. Finally, I wish you and the review experts a happy life and a smooth work ! Thank you again !

(i) The title of section 2.2.3 should include terms 'fluid flow' or 'fluid dynamics' such as 'Governing equations for fluid flow ' (instead of Mechanical equation). Similarly 'Control equation', section 2.2.4, should be renamed e.g. Turbulence modeling.

Reply : The titles of sections 2.2.3 and 2.2.4 have been changed as required.

(ii) HDPE should be deleted from the title as its properties (density, tensile strength etc.) are not used in simulations.

Reply : HDPE in the title of the article has been deleted.
